# Photocatalytic Degradation of Paracetamol in Aqueous Medium Using TiO_2_ Prepared by the Sol–Gel Method

**DOI:** 10.3390/molecules27092904

**Published:** 2022-05-02

**Authors:** Raquel Trujillano, Vicente Rives, Inés García

**Affiliations:** Departamento de Química Inorgánica, Universidad de Salamanca, 37008 Salamanca, Spain; id00691969@usal.es

**Keywords:** titania photocatalysts, sol–gel method, photodegradation of paracetamol

## Abstract

Two titania photocatalysts have been prepared using the sol–gel method using TiCl_4_ as a precursor, and two different alcohols, namely, ethanol or propanol (Et or Pr). The main aim of this work was to study the effect of the nature of the alcohol on the chemical, structural and photocatalytic properties for paracetamol photodegradation of the final solids. The TiCl_4_/alcohol molar ratio to obtain the corresponding alkoxides (TiEt and TiPr) was 1/10. These alkoxides were calcined at 400 °C to prepare the oxide catalysts (named as TiEt400 and TiPr400). Powder X-ray diffraction (PXRD) of the original samples showed the presence of anatase diffraction peaks in sample TiPr, while TiEt is a completely amorphous material. Contrary to commercial TiO_2_-P25, the PXRD diagrams of the calcined samples showed anatase as the exclusive crystalline phase in both solids. The specific surface area (S_BET_) of sample TiPr400 was larger than that of sample TiEt400, and both larger than that of TiO_2_-P25. The three solids have been tested in the photodegradation of paracetamol in aqueous solution. It has been established that the alcohol used influences the properties and catalytic activity of the final oxides. The synthesized solids exhibit a higher activity than commercial TiO_2_-P25, because of their structural characteristics and larger S_BET_.

## 1. Introduction

For some years, there has been a great concern about the presence of emerging contaminants in the environment, since most of the consequences that these compounds can cause and their persistence in the environment are in many cases unknown. Among all the emerging contaminants, some of the most worrying ones are drugs that, due to their wide use by humans, are introduced into ecosystems. Traces of drugs and their metabolites have been found in wastewater, surface and deep water, soil and air, etc., mainly due to the consumption and excretion of drugs (or their metabolites) and the inadequate disposal of unconsumed drugs. The effects of drugs on aquatic organisms have been widely observed by several authors [1,2]. Rossi-Marshall et al. published in 2012 a comprehensive review about pharmaceutical compounds and ecosystem function [3].

In addition, urban, hospital, industrial wastewater, and those of agricultural or livestock origin, are a way for drugs to enter into the aquatic environment. However, what is really worrying about the presence of these pollutants (and mixtures of them) is not only the quantity in which they are found, but also their persistence in the environment, since prolonged exposure has been scarcely studied [4].

Paracetamol is a frequently used analgesic and antipyretic drug. It enters the environment through unchanged excretion in urine and feces or after undergoing transformations producing toxic metabolites (like *N*-Acetyl-*p*-benzoquinone imine, NAPQ). These metabolites pass into the food chain through the aqueous medium [5]. Several studies have shown chronic effects in aquatic organisms due to exposure to paracetamol for long periods of time, affecting their growth rate or reproduction rates [6,7].

Traces of paracetamol have been found in drinking water, which can lead a person exceeding the recommended daily intake [8]. In order to reduce drug-contamination, measures have been proposed, such as legislative changes, reinforcing ERA (Environmental Risk Assessment) or limiting emissions, including drugs as hazardous wastes [9]. Even so, these measures are not enough, and emerging pollutants accumulate in the environment. For this reason, Advanced Oxidation Processes (AOPs) capable of degrading soluble organic pollutants, have been developed and applied in wastewater treatment plants [10,11]. AOPs are characterized by the production of hydroxyl radicals, which are capable of oxidizing almost any organic molecule, producing CO_2_ and H_2_O. Moreover, there have recently been numerous approaches to improve the photocatalytic processes by using TiO_2_-based materials [12,13].

Titania has a strong photo-oxidation power and is one of the major heterogeneous photocatalysts used, mainly because of it is biologically and chemically inertness [14,15]. Brookite, anatase, and rutile are the more known polymorphs of titania. Their structure is based on different packing of [TiO_6_] octahedrals, linked together by sharing edges or corners. The thermal stability of rutile and anatase is higher than that of Brookite and for this reason only they are almost exclusively used for photocatalytic applications [16].

Fernandes et al. have tested TiO_2_ under UV (Ultraviolet) light in order to study the synergistic effect of TiO_2_ used in photocatalytic AOPs in the treatment of refinery effluents [17]. Photocatalytic reactions on TiO_2_ powders are very significant and have been widely studied; actually, titania is the most widely photocatalysts used, due to their applicability to the treatment of a diversity of pollutants [18,19,20], and in chemical conversion of solar energy [21,22]. In these reactions, electrons and holes photogenerated in TiO_2_ particles migrate to the surface, to reduce and oxidize compounds existing in water and air [23].

A large number of papers have described the synthesis procedures and the effectiveness of TiO_2_ as a photocatalyst for the degradation of different contaminants as dyes or phenolic molecules such as paracetamol. For instance, concerning titania nanotubes, López-Zavala et al. have reported [24] that hydrothermal treatment has a deep effect on the crystallinity of the precursor nanoparticles; the purity was highly improved upon acid washing of the particles. They also observed an important effect of the annealing temperature, the highest stable tubular morphology was attained upon annealing at 400–600 °C, but using higher temperatures led to formation of irregular structures. As for the energy of the band gap, that of nanotubes annealed at 400 °C was similar to that of bulk anatase (3.2 eV).

Elizalde-González et al. prepared TiO_2_ nanobelts (TNB) anchored to carbon particles made from soybean husk, and studied the photodegradation and adsorption decolorization of a dye Basic Yellow 28 solution; these authors concluded that the hydroxylated hydrazono-indolium species, and small molecules formed photocatalytically by the TNB, get adsorbed on the carbonaceous portions of the composite [25]. Rimoldi et al. studied the role played by different TiO_2_ properties on the photocatalytic degradation of paracetamol [26]. Jallouli et al. studied the reaction using commercial TiO_2_-P25 (Degussa, Germany) as a photocatalyst in water suspension and under UV light. They studied the effect of pH, concluding that pH 9.0 is optimal under these conditions for drug degradation and that under UV illumination, TiO_2_ is more efficient than TiO_2_/cellulose fiber under sunlight [27]. Suryawanshi et al. studied the degradation of paracetamol, showing that, under the conditions in which they carried out their work, as the pH decreases the rate of paracetamol photodegradation increased [28]. Lozano et al. used TiO_2_ nanotubes for paracetamol photo-degradation and studied the effect of the pH on the on the photoreaction rate [29]. Puri et al. have tested in this reaction a Fe-TiO_2_ composite photocatalyst with a dual effect (photocatalysis and photo-Fenton) [30]. Covaliu et al. described the fabrication of electrospun-based TiO_2_ fibers, and their application in the degradation of organic pollutants, including paracetamol [31].

The aim of this work is to test the effect of using two different alcohols, ethanol (Et) and Propanol (Pr) in the synthesis of TiO_2_ by the sol–gel method described by Zhu et al. [32], on the properties of the titania solids obtained upon calcination, and to compare their textural properties, crystallinity and photocatalytic activity with those of commercial TiO_2_-P25. The chemical, structural, and textural characteristics will be studied by means of the typical characterization techniques for inorganic solids described in Section 3.2.2. The degradation studies have been done by following the photodegradation of an aqueous solution of paracetamol irradiated with UV light and using the solids prepared as photocatalysts. The approach of the work is to have a global study of the effect of the nature of the precursors.

## 2. Results and Discussion

### 2.1. Powder X-ray Difraction (PXRD)

**Figure 1:**
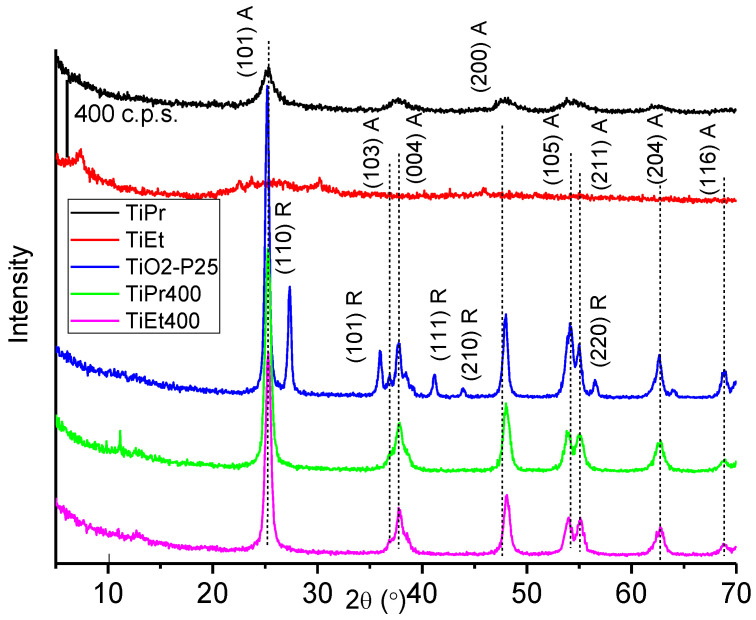
Powder X-ray diffractograms of samples TiPr, TiEt, TiPr400, TiEt400 and of TiO_2_-P25.

The PXRD diagrams of both precursors and both oxide samples are included in Figure 1 where that of TiO_2_-P25 is also included for comparison. The PXRD pattern of TiEt corresponds to that of an essentially amorphous material, but in that of sample TiPr, weak peaks corresponding to the anatase phase of TiO_2_ [33] (Joint Committee for Powder Diffraction Standards card, JCPDS 21-1272) were recorded at 25.4, 37.5, 48.2, 55.2 and 62.7° (2θ) (3.50, 2.39, 1.89, 1.66, and 1.48 Å, respectively).

For the calcined samples, TiEt400 and TiPr400, the diffraction peaks (Figure 1) were recorded at 25.3, 37.9, 48.1, 54.0, 62.8 and 69.0° (2θ) (3.51, 2.37, 1.89, 1.69, 1.48 and 1.36 Å, respectively). The diagrams are almost identical to each other, concerning the positions and relative intensities of the diffraction peaks. In both cases the only crystalline phase identified was anatase. On the other hand, commercial titanium oxide TiO_2_-P25 is known to be composed of approximately 80% anatase 20% rutile (JCPDS 21-1276) [34,35], although these value can change from one batch to another. The diffraction peaks of TiO_2_-P25 are more intense and narrower than those of the TiEt400 and TiPr400 samples, suggesting a larger crystallinity of the former.

The average crystallite sizes of the oxides along the crystallographic direction (101) (which diffraction corresponds to the most intense peak of the anatase phase) have been calculated from the full width at half maximum (FWHM) of this peak, by using the Scherer equation, for the oxides and the reference materials. The crystallite size of commercial TiO_2_-P25 was 18 nm, while for both oxides prepared in this study it was 13 nm, so there was no effect of the alcohol used during the synthesis on the crystallographic properties of the oxides prepared. This result is also consistent with the recorded X-ray diffractograms, since the diffraction peaks of TiO_2_-P25 are more intense and symmetric, indicating a greater crystallinity, in agreement with a larger crystallite size.

### 2.2. FT-IR Spectroscopy (FT-IR)

**Figure 2:**
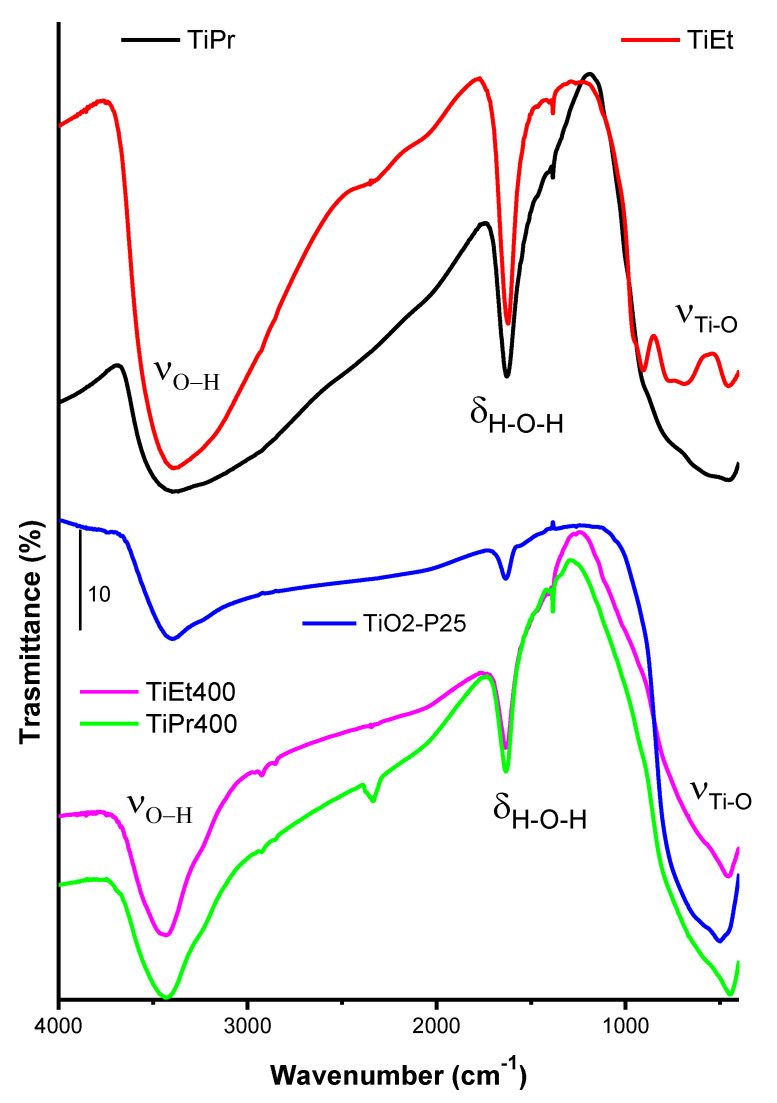
FT-IR spectra of the precursor alkoxides (**top**), the calcined solids TiEt400 and TiPr400 (**bottom**) and of TiO_2_-P25 (**middle**).

The FT-IR spectra of precursor alkoxides TiEt, TiPr, the calcined samples TiEt400, TiPr400, and TiO_2_-P25 are included in Figure 2. The broad and asymmetric band at ca. 3400 cm^−1^ is due to the overlapping of the bands due to the normal stretching modes of different O-H groups existing on the surface of the solid, and of the water molecules [32], linked by hydrogen bonds. The rather intense band at 1620 cm^−1^ is due to the bending mode of water molecules. As these samples have not been calcined, the intensities of the bands due to the water molecules (both bending and stretching modes) are very intense.

The bands in the low wavenumbers region correspond to the Ti-O, Ti-O-Ti and Ti-OH stretching vibrations. For TiPr, there is a single broad band in this range that can be ascribed to the vibrational mode of Ti-OH units [36,37].

The FT-IR spectra of the calcined samples TiEt400, TiPr400 and of TiO_2_-P25 showed the bands at 3400 and 1620 cm^−1^ due to the stretching vibrations of the O-H groups and the deformation mode of the water molecules. They were less intense and broad than in the precursors since during calcination a certain amount of surface hydroxyl groups have been removed (by condensation and elimination of water), as well as the adsorbed molecular water, although this has been partially recovered by exposing the solids to the atmosphere during handling. The very weak bands just below 3000 cm^−1^ in the spectra of samples TiEt400 and TiPr400 are due to C-H stretching modes of adsorbed hydrocarbons from the laboratory environment. The weak band around 2200 cm^−1^ is due to miscancellation of the atmospheric CO_2_ band and the very sharp and weak band at 1390 cm^−1^ recorded in some spectra is due to a minor nitrate impurity in the KBr used to dilute the samples. The FT-IR spectrum of the reference material TiO_2_-P25 shows sharper signals. This confirms, in agreement with the PXRD results, that the reference sample has a higher crystallinity than the samples synthesized and calcined in this work. The three FT-IR spectra show an intense and broad band centered on 400 cm^−1^ associated to the Ti-O stretching vibration of the crystal lattice.

### 2.3. N_2_ Adsorption-Desorption Isotherms

**Figure 3:**
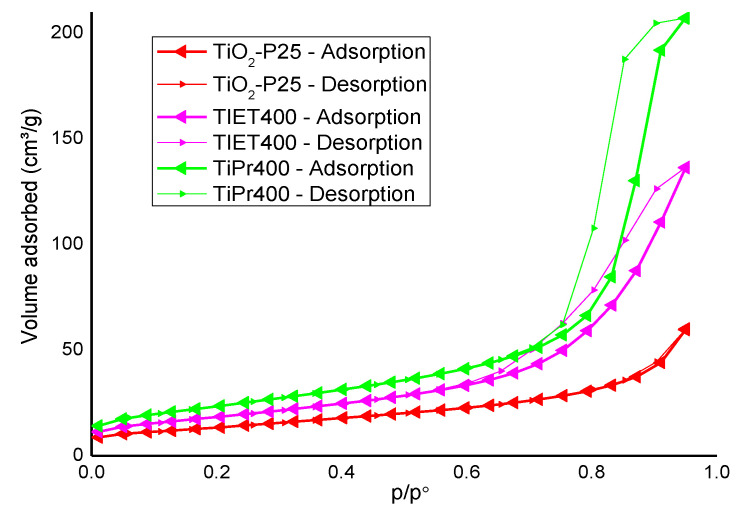
N_2_ adsorption-desorption isotherms of the calcined samples and that of TiO_2_-P25 as comparison.

The textural study of the calcined samples and TiO_2_-P25 was carried out from the adsorption-desorption isotherms of N_2_ at −196 °C. Figure 3 shows the adsorption-desorption curves for the calcined samples TiEt400, TiPr400 and for commercial TiO_2_-P25.

The isotherms of the synthesized samples correspond to type IV in the International Union of Pure and Applied Chemistry (IUPAC) classification [38]. This type of isotherms is typical of adsorption on mesoporous solids (with pores with a diameter between 2 and 50 nm) with multilayer adsorption. This is due to the phenomenon of capillary condensation in the pores on the surface of the solid. The isotherms for both synthetized samples show hysteresis loops corresponding to type H2 according to the IUPAC classification [38]. This hysteresis loops are rather broad and with a small *plateau*, which is characteristic of the presence of pores with a bottleneck shape. This pore geometry, which has a narrow outlet and a wide body, causes the emptying of the pore to be delayed during desorption, forming the *plateau*.

Unlike the previous ones, the N_2_ adsorption-desorption isotherm of TiO_2_-P25 corresponds to type II according again to the IUPAC classification. This type of isotherms is characteristic of non-porous or macroporous solids, that is, with low or null microporosity and with multilayer adsorption without restriction. In this case, the hysteresis loop of the isotherm is so narrow that the process can be considered reversible.

Table 1 summarizes the values for specific surface area, S_BET_ [39], external surface area S_t_ [40], total volume, and pore size provided by the Gemini VII software. As it can be expected from the isotherm, the TiPr400 sample has a higher specific surface area, probably due to the longer chain of the alcohol used to prepare the precursor, creating more and wider channels when it (or its combustion products) diffuses to the surface during calcination and oxide formation.

The S_BET_ values increase as P25 < TiEt400 < TiPr400. For each sample, the values of S_BET_ and S_t_ are almost coincident, thus suggesting the absence of micropores. The pore volumes are significantly different from one sample to another, so the differences in the specific surface areas can be related to the differences in the pore volumes.

### 2.4. Thermogravimetric and Differential Thermal Analysis (TG and DTA)

**Figure 4:**
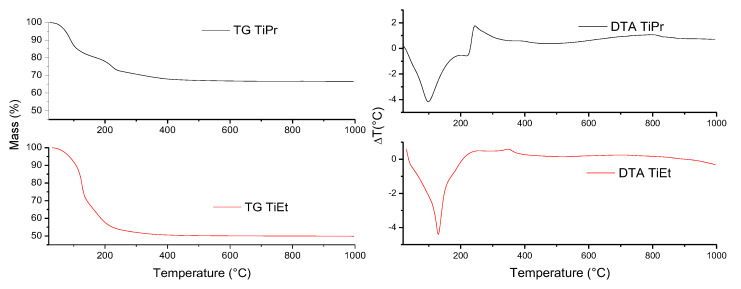
TG and DTA curves of samples TiEt and TiPr.

The TG and DTA curves of samples TiEt and TiPr are included in Figure 4. From the expected formula of the initial compounds, Ti(OC_2_H_5_)_4_ and Ti(OC_3_H_7_)_4_, and of the final compound (which nature, TiO_2_, has been concluded from PXRD analysis, Figure 1), the expected total mass losses were 65 and 71%, respectively, for TiEt and TiPr. The FT-IR spectra in Figure 2 suggest a large water content in these samples which have been merely heated at 70 °C, so the mass losses expected should be even larger [32].

However, according to the TG results, the experimental mass losses were only 50 and 35%, respectively, for samples TiEt and TiPr. These results suggest that the starting compounds were not pure alkoxides, as already confirmed by the PXRD diagram of sample TiPr. This PXRD diagram showed the most intense characteristic diffraction peak of anatase and, if formation of the oxide has been already taken place, then the mass losses recorded would be lower than the calculated ones. In the case of precursor TiEt formation of crystalline anatase was not observed, but we cannot ignore formation of amorphous anatase which would not be detected by PXRD and would account for the TG results obtained.

The TG curve of sample TiEt shows two inflection points close to 135 and 235 °C, coincident with a sharp, intense, endothermic effect in the DTA curve, suggesting that the process associated to these effects should be removal of water and of alcohol molecules [32]. The slope of the TG curve decreases very smoothly until 450 °C and at this temperature the sample has lost 50% of its initial mass [41]. The DTA curve for sample TiEt shows a weak, broad, exothermic effect centered around 350 °C, due to the reorganization of the atoms after decomposition of the precursor, forming a well crystallized anatase phase, as identified by PXRD for the sample calcined at 400 °C. In this temperature range, the combustion of the organic part also takes place, therefore both exothermic effects should be overlapped. It should be highlighted that no strong exothermic effect, associated to combustion of the organic moieties, is recorded, suggesting that at least a significant portion of the alkoxides is decomposed and the alcohol removed before burning. At higher temperatures the mass stabilizes [42,43].

The curves for sample TiPr are somewhat similar, although some effects are much more evident. So, up to three consecutives, overlapped, mass losses can be identified; the associated endothermic effect in the DTA curve is somewhat broader. A sharp exothermic effect is recorded close to 250 °C, suggesting that in this sample propanol burns before or at the same time that it is released [44,45]. A weak, broad, exothermic effect is recorded around 385 °C, which might be ascribed to crystallization of anatase, as identified by PXRD.

**Figure 5:**
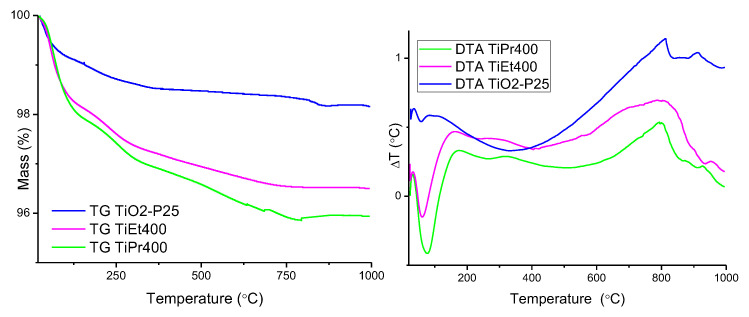
TG and DTA curves of TiEt400, TiPr400 and TiO_2_-P25.

The TG and DTA curves of the oxides (TiEt400, TiPr400 and TiO_2_-P25) are included in Figure 5. The curves are similar for the two calcined samples. All three samples behave similarly; the total mass loss corresponds to less than 5% of the original mass sample. Previous calcination at 400 °C to prepare these oxides has completely removed the organic groups of the alkoxide precursors and has partially dehydroxylated the titania surfaces. So, the water content (in the form of surface hydroxyl groups and of adsorbed water molecules) should be rather small, and even some of the water existing on the sample surface might be due to reabsorbed water from the atmosphere during handling of the samples. Nevertheless, the TG curve shows in all three cases a first, sharp mass loss probably due to removal of water, while the second mass loss, with a smaller slope in the TG curve, is probably due to removal of surface hydroxyl groups, which condense forming water molecules. Some “waves” in the DTA curve would be associated to these effects, i. e., and not all hydroxyl groups are removed in the same temperature range. Probably, the strength of the Ti-OH bonds depends on the specific nature of the crystalline faces exposed, thus accounting for such a loss of hydroxyl groups in several consecutive steps. In all cases, a strong, ill-defined, broad, exothermic effect is recorded above 600 °C, extending for more than 200 °C, which probably corresponds to transformation of anatase into rutile, the most stable titania phase at high temperature [46].

### 2.5. Photocatalytic Activity

To assess the photocatalytic activity, the paracetamol degradation reaction was studied in a UV quartz reactor in the presence of TiEt400, TiPr400 or TiO_2_-P25 as photocatalysts. The progress of the reaction was followed by recording the UV spectrum of the solution after selected reaction times. Paracetamol shows in its UV spectrum two characteristic absorption bands with λ_max_ at 208 nm and 243 nm corresponding to electronic π–π* and n–π* transitions, respectively, of the C=O group [47]. The decrease in the intensity of the band recorded at 243 nm was chosen to check photodegradation of paracetamol.

At the beginning of the experiment with each catalyst (or for the photolysis reaction), the suspension was kept in the darkness and aliquots were taken at set times until the paracetamol concentration stabilized and remained constant. This step was taken because the concentration of paracetamol can somewhat decrease due to its adsorption on the TiO_2_ surface. Once the paracetamol concentration remained constant, the ultraviolet lamp was turned on and aliquots continued to be taken. These aliquots, as the previous ones, were kept in the darkness until they were filtered with a syringe filter and analyzed in the UV-Vis spectrophotometer. To check if the photolysis reaction takes place, the solution was submitted to stirring under UV radiation from the beginning (as obviously no adsorption of paracetamol on any catalyst was expected) and aliquots were taken periodically and did not need to be filtered since there was no solid catalyst in suspension.

**Figure 6:**
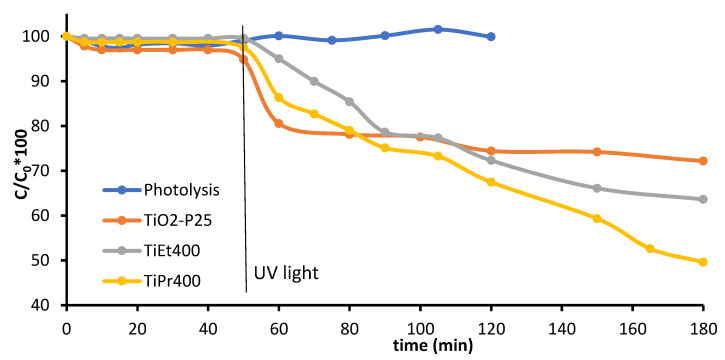
Curves of degradation % vs. the reaction time of the samples. Photolysis and results for TiO_2_-P25 are included for comparison. The vertical line indicates switch on of the UV lamp.

The result of the photolysis and photodegradation process can be seen in Figure 6, where the relative concentration of the contaminant (C_0_ = initial paracetamol concentration; C = concentration at a given time), calculated from the intensity of its band at 243 nm, is plotted vs. the reaction time, up to ca. 2 h of reaction. The concentration of paracetamol did not change significantly in the absence of the catalyst, so we must conclude that there was no photolysis.

During the time the solids tested were suspended in the solution in the darkness, the decrease in paracetamol concentration in the presence of the catalysts was null or negligible; adsorption of paracetamol was thus negligible. It can be concluded that paracetamol degradation was almost entirely due to the photo-activity of the catalysts. However, the behavior of the three catalysts showed some differences.

Once the system was illuminated with UV light, the concentration of paracetamol decreased sharply for commercial TiO_2_-P25, but such a decrease was slower for catalyst TiPr400 and even slower for catalyst TiEt400. The decreases after 10 min reaction were 20, 15, and 5%, respectively, of the initial paracetamol concentration. From this time on, the amount of paracetamol photodegraded on TiO_2_-P25 remained almost constant, even after 2 h under UV light. However, photodegradation in the presence of the synthetized catalysts continued, but faster on catalyst TiPr400 than on catalyst TiEt400. The origin of the difference in this behavior among the three photocatalysts tested is not clear, but the different photo-activity of commercial TiO_2_-P25 can be tentatively ascribed to the fact that it contains both anatase and rutile, while those photocatalysts here synthetized are formed entirely of anatase [48,49,50].

**Figure 7:**
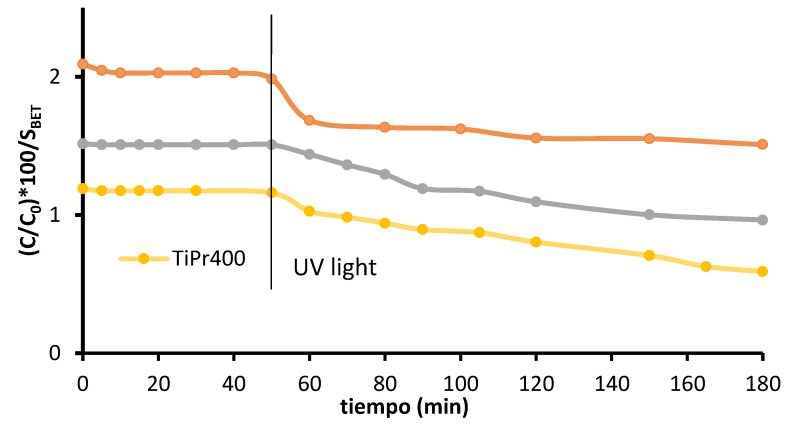
Curves of degradation %/S_BET_ vs. time. The vertical line indicates switch on of the UV lamp.

To highlight the importance of obtaining solids with a high specific surface, the results of the catalytic activity have been related to the specific surface area of the photocatalysts, since heterogeneous catalysis is a phenomenon that occurs on the surface of the solid. To study the incidence of S_BET_ values on the photocatalytic activity, the relative concentration percentage of paracetamol per surface are unit versus the reaction time with each of the photocatalysts tested has been plotted in Figure 7. As it can be seen, the relative concentration of paracetamol per surface area unit throughout the measured reaction time was higher when using TiO_2_-P25, followed by TiEt400, but lower when using TiPr400. These data confirm that the higher photo-activity shown by sample TiPr400 is due to its higher specific surface area.

### 2.6. Kinetics Study of Photocatalytic Degradation

The Langmuir-Hinshelwood model [51] can be used to describe the relationship between the rates of the photocatalytic degradation of paracetamol in the presence of TiEt400, TiPr400 and TiO_2_-P25 as a function of irradiation time. The rate equation of this model is [52,53]:

(−dC/dt) = (k_L−H_·K_ad_C)/(1 + K_ad_C)

Assuming that the adsorption coefficient K_ad_ is <<1, for the pseudo-first-order reaction the equation can be simplified to

Ln(C_0_/C) = kt

where k = k_L–H_·K_ad_ is the pseudo-first-order reaction rate constant, and the half-life time t_1/2_ can be calculated as t_1/2_ = Ln(2)/k. These kinetic parameters, calculated from the Ln(C_o_/C) vs. time plots, are included in Table 2 and confirm the best role played by catalyst TiPr400 for paracetamol photodegradation.

## 3. Materials and Methods

### 3.1. Materials

The reagents used to prepare and to characterize the compounds are summarized in Table 3. The supplier companies are also included in the table.

### 3.2. Methods

#### 3.2.1. Synthesis Methods

These titanium oxide photocatalysts have been obtained using the sol–gel method described by Zhu et al., in which TiCl_4_ is used as a precursor [23]. In this work, two solvents have been used to carry out the syntheses and to obtain the corresponding alkoxides, which were subsequently calcined to obtain TiO_2_.

##### Synthesis of Ti(OEt)_4_

An amount of 50 mL of ethanol were placed in a beaker with a magnetic stirrer and 5 mL of TiCl_4_ were dropwise added from a separation funnel. Once the addition was complete, a yellow solution of Ti(OEt)_4_ was formed, which was left standing for 24 h without stirring, allowing polymerization. The mixture was gently (70 °C) heated for 5 h in a water bath until a gel was formed and ethanol was slowly evaporated. To completely dry the solid, the mixture was placed in an oven at 45 °C overnight. The solid formed was named as TiEt, and it was calcined in a furnace at 400 °C for 1 h in air at using a heating rate of 10 °C/min. The solid obtained after calcination has been designated as TiEt400. The yield was 61%, from the initial amount of TiCl_4_ used.

A similar procedure was followed to prepare TiPr400 from TiCl_4_ and n-propanol, with a yield of 69%.

#### 3.2.2. Instrumentation

The powder X-ray diffraction (PXRD) diagrams of the samples have been recorded from 5 to 70° (2θ) at 2°/min, using a Siemens D-5000 diffractometer, operating at 40 kV and 30 mA, and using filtered Cu Kα radiation (λ = 1.5418 Å). The FT-IR spectra were recorded in the 4000–450 cm^−1^ range in a Perkin Elmer Spectrum-One spectrometer by the KBr pellet method. The textural properties were determined from the nitrogen adsorption–desorption isotherms recorded at −196 °C, using a Micrometrics Gemini VII 2390t, Surface Area and Porosity apparatus. The thermogravimetric and differential thermal analyses (TG, DTA) curves were recorded on a SDT Q600 TA instrument, under an oxygen flow of 20 mL/min of and a heating rate of 10 °C/min from room temperature (RT) to 1000 °C.

#### 3.2.3. Photodegradation Studies

A MPDS-Basic system from Peschl Ultraviolet, with a PhotoLAB Batch-L reactor and a TQ150-Z0 lamp (power 150 W), integrated in a photon CABINET, was used to check the photo-activity. Its spectrum is continuous, with the main peaks at 366 nm (radiation flux, 6.4 W) and 313 nm (4.3 W).

All the solutions were analyzed by Visible-Ultraviolet spectroscopy to control the progress of the reaction. The Vis-UV spectra were recorded in a Perkin-Elmer LAMBDA 35 spectrophotometer coupled to a computer using UV WINLAB 2.85 software [54].

For each analysis, one gram of TiO_2_ (synthetized sample or commercial TiO_2_-P25) was added to 750 mL of an aqueous solution of paracetamol (50 ppm), that is, a 1.33 g catalyst/L ratio. The suspension was continuously stirred within the chamber under illumination with a UV quartz lamp. The reactor was cooled with a water flow and the temperature fixed at 25 °C. For comparison, the photodegradation under UV light, in the same conditions, but in the absence of a catalyst (photolysis) was also studied.

## 4. Conclusions

The method used has led to formation of titanium dioxide with the anatase structure, as confirmed by PXRD. The FT-IR analysis and the results of the DTA and TG thermal analysis confirm the results found by X-ray diffraction. The use of different starting alcohols has led to formation of titania powders with different textural features.

The synthesized solids have been tested in the photodegradation of paracetamol in aqueous solution, and their activity has been compared to that of TiO_2_-P25, a widely used commercial photocatalyst. The synthesized solids show a higher photocatalytic activity than TiO_2_-P25, due to the different physicochemical properties of the solids, probably due to the synthesis method used, that allows to obtain quickly, economically and efficiently TiO_2_ with the anatase structure and with a higher specific surface area than the commercial catalyst.

It can be concluded that the photocatalytic behavior of the solids tested depends on the value of their specific surface area and, with the results found, the importance of the synthesis method in obtaining solids conceived for their use in heterogeneous photocatalysis reactions, such as paracetamol photodegradation, has been highlighted.

Photocatalyst TiPr400 shows the best behavior, probably due to its textural properties (as the chemical composition is the same in all three cases). This fact is probably related to the longer hydrocarbon chain of the precursor used to prepare this photocatalyst which, upon thermal decomposition, gives rise to a larger amount of gases and vapors, thus accounting for the larger specific surface area and pore structure; probably, the surface concentration of hydroxyl groups is also larger, and thus a larger concentration of OH**^·^** radicals (active species in the photodegradation reaction) will be formed upon photoexcitation. The exclusive presence of anatase, with an indirect band gap, in the samples prepared in this study, contrary to the mixture of anatase and rutile (with a direct band gap), might also account for the differences observed. These findings open new perspectives to develop optimum photocatalysts, by using alcohols with larger hydrocarbon chains or even branched-chain hydrocarbons during the synthesis of the precursors.

## Figures and Tables

**Table 1:** Textural parameter values of the calcined samples and TiO2-P25.

	S_BET_ (m^2^/g)	S_t_ (m^2^/g)	Volume_pore_ (cm^3^/g)	Size_pore_ (Å)
TiEt400	66	68	0.212	102
TiPr400	84	83	0.321	103
P25	48	48	0.094	77

**Table 2:** Kinetic parameters.

	TiEt400	TiPr400	TiO_2_-P25
**k (min^−1^)**	0.0041	0.0056	0.0024
**t_1/2_ (min)**	169	124	289

**Table 3:** Reagents and gases used and their supplier companies.

Formula	Chemical Name	Supplier
TiO_2_ (P25)	Titanium dioxide	Degussa
Paracetamol	*N*-(4-hydroxy phenyl) acetamide	Kern Pharma
TiCl_4_	Titanium (IV) Chloride	Merck KGaA
CH_3_CH_2_OH	Ethanol absolute	Panreac
CH_3_CH_2_CH_2_OH	1-propanol (99.5%)	Panreac
KBr	Potasium bromide (for FTIR spectroscopy)	Panreac
N_2_ (liquid)	Liquid nitrogen	L’Air Liquide España S.A.
N_2_	Nitrogen gas, N-35 (99.95%)	L’Air Liquide España S.A.
O_2_	Oxygen N-45 (99.995%)	L’Air Liquide España S.A.
He	Helium, N-48 (99.998%)	L’Air Liquide España S.A.

## Data Availability

All data generated or analyzed during this study are included in this article.

## Sample Availability

Samples of the compounds are not available from the authors.
